# Protecting vaccine confidence: a core function of national immunization programs

**DOI:** 10.3389/phrs.2026.1610300

**Published:** 2026-09-24

**Authors:** Margarida Tavares

**Affiliations:** 1 EPIUnit ITR, Instituto de Saúde Pública da Universidade do Porto, Universidade do Porto, Porto, Portugal; 2 Department of Infectious Diseases, Unidade Local de Saúde de São João, Porto, Portugal

**Keywords:** diphtheria, immunization programs, infodemic management, public health governance, vaccination mandates, vaccine advisory committees, vaccine confidence, vaccine hesitancy

## Introduction

In the early hours of 20 August 2026, a four-year-old girl died of diphtheria in Palermo, the first fatal case reported in Italy in more than three decades. According to reports, she and her three siblings had not been vaccinated because their parents believed that a vaccine had caused autism in a close relative. A vaccinated cousin of the same age, hospitalized with the same infection, had only mild disease. The girl had initially been assessed in an emergency department and discharged with a diagnosis of exudative tonsillitis before being readmitted as her condition deteriorated^1^. The death led to investigations into both the child’s non-vaccination and the medical care she received.

Cases such as this are often interpreted primarily as the consequence of individual parental decisions. That interpretation might not be wrong, but it is incomplete. This case did not result from lack of access to vaccination. It appears to have resulted from a belief that kept four children unvaccinated for years, in a country where diphtheria vaccination is legally mandatory. Individual decisions matter, but so do the responsibilities of public health institutions. The relevant question extends beyond why one family refused vaccination, to what immunization policies, and those who implement them, can do differently for all recommended vaccines.

## From individual hesitancy to institutional responsibility

For the past two decades, vaccine hesitancy — the delay or refusal of vaccination despite the availability of services, as defined by WHO’s Strategic Advisory Group of Experts (SAGE) [1] — has been the dominant framework for understanding declining vaccine uptake. It replaced a crude “informed versus misinformed” dichotomy with a continuum between unquestioning acceptance and outright refusal and showed that most vaccine-hesitant individuals are not opposed to vaccination but uncertain about it, delaying some vaccines while accepting others. Behavioral models such as WHO’s Behavioral and Social Drivers of Vaccination (BeSD) [2] and the 5C model [3] have since enabled a more systematic understanding of the behavioral and social determinants of vaccination. Yet the claim that vaccines cause autism, originating from a fraudulent publication that was later retracted, continues to influence vaccination decisions more than two decades later [4, 5], illustrating that debunking misinformation is not enough to restore confidence.

A previous commentary in Public Health Reviews argued that Europe lacks a shared framework for tackling vaccine hesitancy at the individual or systemic level [6]. We take the second view: the hesitancy framework explains how people decide, but says little about what health authorities owe families before that decision hardens into non-vaccination. Palermo illustrates that limitation. We do not know what opportunities for dialogue or intervention existed for this family, or whether any were missed. But the case raises a question that extends well beyond one household: whatever their institutional form, what responsibilities do immunization programs have to identify, engage and support families before non-vaccination results in serious illness? The quality of an immunization program should not be judged solely by the vaccination coverage it achieves, but also by its capacity to recognize these early signals and respond before preventable disease occurs.

## Confidence has a measurable cost

The COVID-19 pandemic made the consequences of declining vaccine confidence visible. In the United States alone, misinformation about COVID-19 vaccines has been estimated to have contributed to approximately 45,000 preventable deaths and more than US$2 billion in hospital costs during 2021 [7]. The pandemic also showed that more communication does not translate into more confidence: health authorities communicated more than ever before, yet an infodemic, the overload of true and false information that makes reliable information hard to find, competed with that communication at an unprecedented scale. Addressing the infodemic became a public health priority, and WHO outlined this approach [8]: monitoring public concerns, information environments and behavioral trends to detect declining confidence before it translates into falling vaccine uptake. The shift is from responding to misinformation once it has already spread, to detecting emerging concerns early enough to act through dialogue, evidence and appropriate public health measures.

## Vaccine confidence as a core programmatic function

Vaccine confidence deserves the same standing in immunization programs as procurement, delivery, surveillance and coverage monitoring. It should not be treated as a communication add-on, summoned only once confidence starts to slip. It needs to be tracked and protected all the time. Coverage is essential, but it is a late indicator; confidence can erode for months or years before it shows up in the figures. Programs that also track the behavioral and social drivers of vaccination, engage with communities and invest in trust before crises arise are better placed to notice that erosion while there is still time to act.

## Beyond the mandate

Treating confidence as a programmatic responsibility does not amount to state paternalism over individual choice. Vaccination against a transmissible disease is not a purely self-regarding decision: an unvaccinated child is not only at risk herself, but a possible link in a chain of transmission to infants too young to be vaccinated, to people who cannot be vaccinated for medical reasons, and to anyone whose protection depends on population-level immunity. That distinction between self-regarding and other-regarding behavior grounds vaccination as a public health measure rather than an individual choice.

Countries differ widely in how they translate this responsibility into law. Some enforce active mandates: Italy expanded its list of compulsory childhood vaccines to ten in 2017, reinforcing existing obligations, including diphtheria, and adding new ones, with penalties for non-compliance [9], and France followed a similar path in 2018, extending its list further since^2^. Others make no vaccines compulsory, relying instead on recommendations issued by technical bodies and implemented through more or less decentralized structures, as in Germany^3^ and Switzerland^4^, where confidence carries more of the weight in the absence of legal obligation. Still others retain vaccination laws that are rarely enforced, leaving uptake to depend almost entirely on public trust. Legal mandates and public confidence are complementary rather than interchangeable: a mandate can raise coverage for the vaccines it targets, but sustaining high uptake over time, and across the vaccines no mandate covers, depends on confidence [5].

One condition for that confidence is the technical independence of the advisory bodies that issue immunization recommendations. In 2025, that independence came under political pressure in both Italy and the United States: an advisory panel was dissolved in Italy over contested appointments^5^ and entirely replaced in the United States amid criticism of politicized decision-making [10].

## Conclusion

The diseases that vaccination prevents have not disappeared, and most cannot realistically be eradicated. They remain merely controlled, for as long as confidence in vaccines and in institutions holds. The first signs of faltering confidence rarely appear in emergency departments. They appear earlier, when institutions trade scientific independence for political convenience, and in communities whose concerns have never been asked about. Waiting for the visible return of disease to act is, almost always, arriving too late. That lesson will only grow more urgent as new and re-emerging infectious threats require rapid, sustained vaccine uptake. Palermo should be remembered not only as an individual tragedy, but as a reminder that protecting vaccine confidence is not a communication task delegated downstream. It is a core governance function of national immunization programs, and it deserves to be resourced, measured, and defended as one.

## Footnotes

1 ANSA. Bimba di 4 anni muore per sospetta difterite a Palermo. 20 August 2026. Available at: https://www.ansa.it/sito/notizie/cronaca/2026/08/20/bimba-di-4-anni-muore-per-sospetta-difterite-a-palermo_88e2de87-751a-4c96-bd7c-517ffd0ff4ff.html

2 Ministère de la Santé, de la Famille, de l'Autonomie et des Personnes handicapées. De nouveaux vaccins obligatoires pour les nourrissons. Available at: https://sante.gouv.fr/prevention-en-sante/preserver-sa-sante/vaccination/vaccins-obligatoires/

3 Federal Ministry of Health (Germany). Vaccinations. Available at: https://www.bundesgesundheitsministerium.de/en/topics/vaccinations

4 Federal Office of Public Health (Switzerland). National Immunisation Programme. Available at: https://www.bag.admin.ch/en/national-immunisation-programme

5 Medscape. Antivaccine voices topple Italy’s vaccine advisory panel. 2025. Available at: https://www.medscape.com/viewarticle/antivaccine-voices-topple-italys-vaccine-advisory-panel-2025a1000mon
